# Benefits of re-do surgery for recurrent intracranial meningiomas

**DOI:** 10.1038/s41598-019-57254-5

**Published:** 2020-01-15

**Authors:** Jean-Michel Lemée, Marco V. Corniola, Torstein R. Meling

**Affiliations:** 1 Department of Neurosurgery, University Hospital of Angers, Angers, France; 2 Department of Clinical Neurosciences, Division of Neurosurgery, Geneva University Hospitals, Geneva, Switzerland; 3 Faculty of Medicine, University of Geneva, Geneva, Switzerland; 4 Department of Neurosurgery, Oslo University Hospital, Oslo, Norway; 5 Faculty of Medicine, University of Oslo, Oslo, Norway

**Keywords:** CNS cancer, Surgical oncology

## Abstract

Meningiomas are the most common intracranial extra-axial tumor. While the literature is abundant on the therapeutic management of meningioma recurrence after the initial surgery, the natural history of repeated recurrences is poorly described, as well as and their respective management. A partly retrospective, partly prospective review was conducted in a Norwegian cohort of 1469 consecutive cases of meningioma surgically treated, totaling 11 414 patient-years of follow-up. 114 recurrences (7.7%) were treated surgically with a risk a surgical retreatment of 1% per patient-year of follow-up. 36 patients were operated on 3 times or more. The time-to-retreatment (TTR) decreased significantly and steadily between surgeries, from 4.3 ± 4 years after the first surgery to 2.4 ± 2.9 years after the third surgery. The primary driver for recurrence was the WHO grade (OR 7.13 [4.40;11.55], p < 0.001 for the first recurrence and OR 4.13 [1.49;12.15], p 0.008 for the second), the second predictive factor being a skull base location (OR 2.76 [1.95;3.99] p < 0.001 and OR 0.24 [0.09;0.65], p0.006 respectively). The rates of postoperative hematomas and infections were not influenced by the number of surgeries, whereas the rate of postoperative neurological worsening increased from 3.9% to 16.6% and 13.9%, respectively, after the first, second, and third surgeries. We observed that the TTR decreased significantly between surgeries in patients requiring repeated resections, indicating that surgical treatment of recurrences does not reset the clock but is indeed a “race against time”. This should be considered when assessing the benefit-to-risk ratio of patients undergoing repeated surgeries for a recurrent meningioma.

## Introduction

Meningiomas are the most frequent intracranial extra-cerebral tumors^1,2^. They are classified according to the World Health Organization (WHO) in histopathological grades from I to III^3^. The therapeutic management is primarily surgical, aimed at a complete resection of the tumor with its dural tail^4^, followed by a clinico-radiological follow-up^5,6^. This “intervention-first” paradigm allows for high rates of disease-control^6,7^.

However, the majority of meningiomas show slow growth, making their therapeutic management similar to a chronic disease with a lengthy clinico-radiological follow-up^8,9^. Should the tumor recur, the treatment is surgical whenever possible, according to the patient’s functional status and Karnofsky Performance Scale (KPS). Stereotactic or conventional radiation therapies are privileged otherwise. Therefore, early recognition of recurrence and its management is of paramount importance for neurosurgeons.

While the literature is abundant on the therapeutic management of meningioma recurrence after the initial surgery, the natural history of repeated recurrences and their therapeutic management is poorly described. Furthermore, there is little information regarding the interval between surgeries (or any treatment) and successive recurrences. Such information would be useful to assess the benefit-to-risk ratio of a re-intervention in the context of a recurrent meningioma.

In this study, we describe the surgical management of re-recurring meningiomas as well as the postoperative complications thereof in a population-based cohort of 1469 patients surgically treated for a meningioma totaling 11 414 patient-years of follow-up.

## Results

### Baseline demographics and general data

A total of 179 patients (12.2% of the total cohort) had tumor recurrences requiring retreatment after a mean time-to-retreatment (TTR) between the first surgery and the first subsequent treatment (either a new surgical procedure or radiotherapy) of 3.6 ± 3.6 years. The female-to-male sex-ratio was 2.4:1. The mean preoperative Karnofsky Performance Score (KPS) was 81.2 ± 12.1. Gross total resection (GTR) was achieved in 78.8% of cases, Simpson grades are reported in Table 1, as well as baseline demographics and neurological symptoms upon admission. From the overall cohort, a total of 43 patients (2.9%) had adjuvant radiation therapy; among the 179 patients with recurrence, 11 patients (6.1%) had an adjuvant radiation therapy (N = 3, 3 and 5 WHO grade I, II and III, respectively).

**Table 1:** Characteristic of the population.

	n	%
Age	58 ± 20.1	—
Sex	1033F/436 M	—
Preoperative KPS	81.18 ± 12.1	—
**Presenting symptoms**		
Asymptomatic	79	5.4%
Seizures	435	29.6%
Raised ICP	466	31.7%
Neurological deficit	855	60.2%
**WHO grade**		
I	1352	92.3%
II	77	5.2%
III	32	2.2%
Skull base tumor	690	47%
Bone infiltration	274	18.7%
**Simpson grade**		
I	575	39.2%
II	503	34.2%
III	79	5.4%
IV	302	20.6%
V	8	0.6%
Adjuvant radiation therapy	43	2.9%
Recurrence	179	12.2%
Surgery	114	7.8%
Radiation therapy	65	4.4%
**Time to retreat at first recurrence**		
All	3.9 ± 3.9	—
Surgical treatment	4.3 ± 4	—
Radiation therapy	2.5 ± 2.9	—
Follow-up (years)	7.8 ± 5.5	—

ICP: intracranial pressure; KPS: Karnofsky performance score; RT: radiation therapy; WHO: world health organization.

### Therapeutic management of recurrent meningiomas

**Figure 1:**
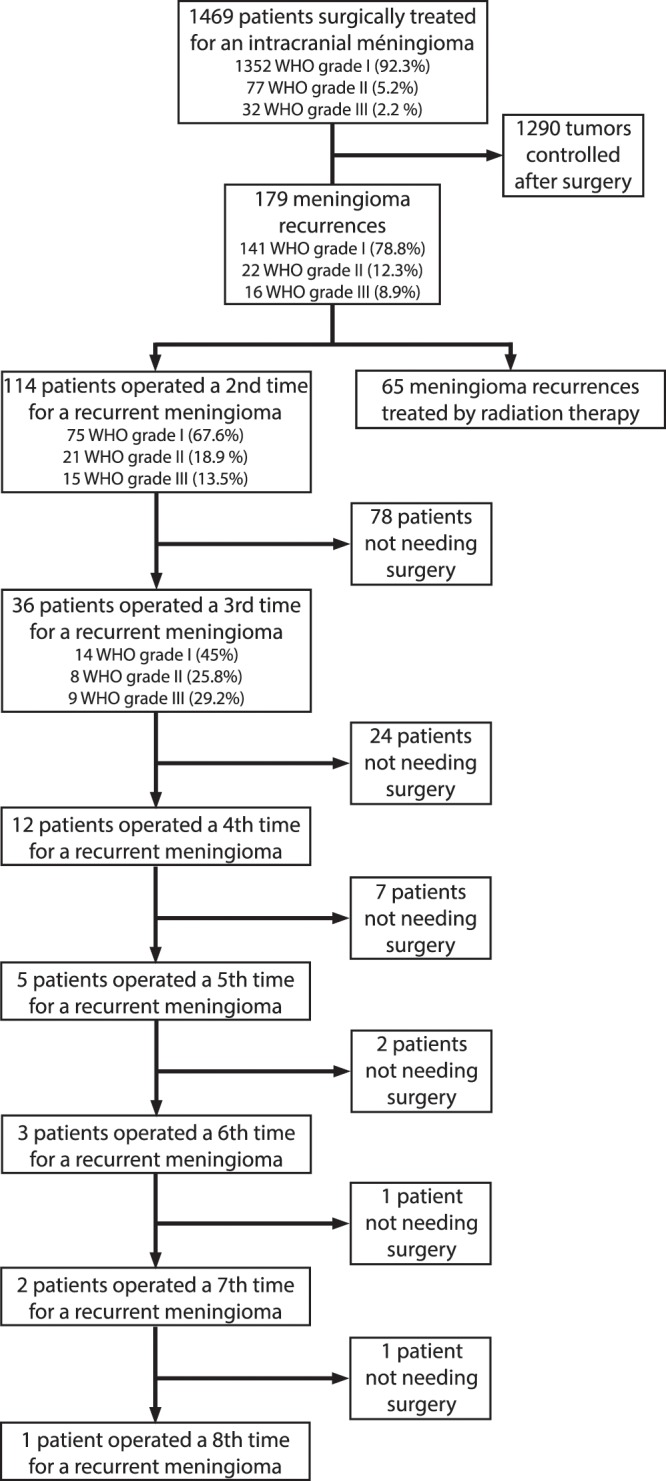
Flow Chart of the therapeutic management or recurrent meningioma in our cohort.

Of the 179 patients treated for a recurrent meningioma, 114 patients (63.7%) had a second surgery and 65 (36.3%) were treated by radiation therapy (Fig. 1). The recurrence rate was of 1.6% per patient-year of follow-up in our cohort of patients surgically treated for an intracranial meningioma. The TTR was significantly longer in recurrent meningioma treated with surgery than in the group treated by radiation therapy (4.3 ± 4.0 vs. 2.5 ± 2.9 years, p < 0.001) (Table 1).

### Surgical management of recurrent meningiomas

**Figure 2:**
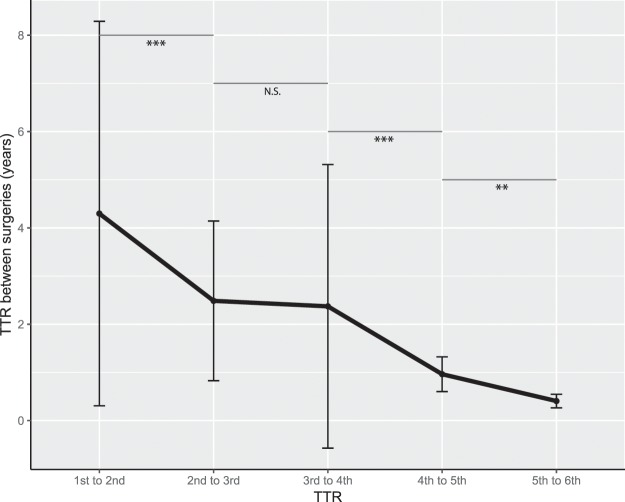
Graphical representation of TTR between surgeries for recurrent meningioma. **p < 0.01. ***p < 0.001. N.S.: not statistically significant.

Of the 114 patients (7.7% of the total cohort) who underwent a second surgery, 95% of patients were symptomatic upon admission. The risk of a second surgery for a recurrence was 1% per patient-year of follow-up in the population treated surgically for an intracranial meningioma. The mean age at surgery was 58.1 ± 20.1 years and the mean TTR was 4.3 ± 4 years. Among these, 36 patients (2.4% of the total cohort) underwent a third surgery with a significantly shorter TTR of 2.5 ± 1.7 years between the 2^nd^ and 3^rd^ surgery (p < 0.001). Twelve patients (0.8% of the total cohort) had a fourth surgery, with a TTR of 2.4 ± 2.9 years between the 3^rd^ and 4^th^ surgery. Five patients (0.3%) had a fifth surgery with a significantly inferior (p < 0.001) TTR of 0.96 ± 0.4 years between the 4^th^ and 5^th^ surgery, and 3 (2%) patients had a sixth surgery with also a significantly inferior (p 0.01) TTR of 0.4 ± 0.1 between the 5^th^ and 6^th^ surgery (Fig. 2). Two patients were operated 7 and 8 times, respectively.

We observed a modification of meningioma’s WHO grade proportions between recurrences, with an increase of WHO grade II and III meningiomas. At the first surgery, WHO grade II and III meningiomas represented 5.2% (n = 77) and 2.2% (n = 32), 18.9% (n = 21) and 13.5% (n = 15), respectively, for the second surgery and 25.8% (n = 8) and 29% (n = 9) for the third recurrence (Fig. 1). The increase of WHO grade II and III meningiomas’ ratio between surgeries was statistically significant for all comparisons (p < 0.001).

### Predictive factors of recurrence

Age was the only patient-related predictive factor of recurrence after first surgery (OR 0.97 [0.96;0.98], p < 0.001). Skull-base location and WHO grade were the two identified tumor-related factors for recurrence (OR 2.76 [1.95;3.99] p < 0.001 and OR 7.13 [4.40;11.55], p < 0.001).

The predictive factors of recurrence after the second surgery were skull-base location (OR 0.24 [0.09;0.65], p = 0.006) and WHO grade (OR 4.13 [1.49;12.15], p = 0.008). Predictive factors of recurrence are summarized in Table 2.

**Table 2:** Predictive factors of recurrence.

	1^st^ recurrence		2^nd^ recurrence	
	OR	p	OR	p
Age	0.97 [0.96; 0.98]	**<0.001**	0.97 [0.94; 1.01]	0.16
Sex (Male)	1.09 [0.75; 1.57]	0.63	0.78 [0.27; 2.14]	0.64
Preoperative Karnofsky ≥70	1.84 [0.89; 4.36]	0.12	0.84 [0.10; 9.18]	0.88
Skull base location	2.76 [1.95; 3.99]	**<0.001**	0.24 [0.09; 0.65]	**0.006**
WHO grade	7.13 [4.40; 11.55]	**<0.001**	4.13 [1.49; 12.15]	**0.008**
Simpson grade	0.99 [0.85; 1.16]	0.98	NA	NA
Postoperative hematoma	0.42 [0.07; 1.54]	0.27	0.42 [0.02; 1.29]	0.57
Postoperative infection	0.66 [0.15; 2.08]	0.54	1.47 [0.16; 3.32]	0.75
Adjuvant radiation therapy	0.75 [0.42; 1.32]	0.34	NA	NA

NA: data not available; RT: radiation therapy; WHO: world health organization.

Because of a low population of patients with a meningioma recurrence treated surgically for the 3^rd^ time, a multivariate analysis of predictive factors of meningioma recurrence was not performed.

### Postoperative complications following surgery for recurrent meningiomas

Postoperative hematoma was identified in three patients (2.7%) after the second surgery and none after the third surgery, compared to a rate of 2.6% after the first surgery. This difference was not statistically significant.

Postoperative infections were recorded in four patients (3.5%) after the second surgery and in one patient (2.7%) after the third surgery, compared to 2.7% after the initial surgery. This difference was not statistically significant.

A total of 19 patients (16.6%) had a postoperative worsening of neurological function after the second surgery and five patients (13.9%) after the third surgery, representing a significant increase compared to 3.9% after the first surgery (p < 0.001).

## Discussion

While the literature on predictive factors and management of meningioma recurrence is abundant, little is known on the natural history of repeated meningioma recurrence, its therapeutic management and its impact on tumor evolution^5,6,10–13^. In this study, based on one of the largest consecutive population-based cohorts to date, we identified the rate of surgical re-intervention for recurrent meningiomas, the predictive factors of recurrence, as well as postoperative outcomes of recurrent surgeries.

Recurrences are relatively rare after meningioma surgery^14^, with a 7.7% rate in our cohort of 1469 patients after a follow-up of 11 414 patient-years. In our study, we observed that the TTR decreases significantly between surgeries in patients requiring repeated resections, indicating that surgical treatment of recurrences does not “reset the clock” but is indeed a “race against time”. In other words, the benefits of a surgical treatment of meningioma recurrences decrease with the number of surgeries, while the risk of complications remains stable. This does not mean that the benefits of surgery are lost in repeated recurrence the benefit-to-risk ratio changes with successive surgeries, which are also more dangerous and more technically challenging. However, repeat surgery is warranted for a recurrence whenever possible and it is of interest to appreciate recurrence and complication rates as well as the TTR after a re-do surgery for meningioma recurrences. This will allow the surgeon to have a better appreciation of the benefit-to-risk ratio of a repeat surgical procedure in order to aid the therapeutic decision, be it surgery, radiation therapy or a simple clinico-radiological follow-up, and to give the patient a better appreciation of the surgical challenges and risks.

In our cohort, we identified 114 patients surgically retreated for a recurrent meningioma after a mean follow-up of 7.8 ± 5.5 years. This represents a 1% risk of surgical retreatment per patient-year of follow-up. Patient- and tumor-characteristics were similar to previous studies reported in the literature^15,16^. However, the recurrence rate was significantly lower than in previously reported data^5,16^. This is especially noteworthy as we have a high proportion of skull-base meningiomas in our overall cohort (47%). Since complete surgical resection is less frequently obtained in skull-base meningiomas, they are more prone to recurrence^17,18^, and the skull-base location of meningioma has previously been identified as a significant negative predictive factor for TTR^19^. Firstly, our results may be explained by the expertise of the tertiary referral center in the management of meningiomas, especially for the skull-base meningiomas. Secondly, the definition of meningioma recurrence in this study may play a role since a radiological recurrence without clinical impact that did not require an adjuvant treatment were not considered.

According to the standard of care, all recurring meningiomas were treated surgically whenever possible considering tumor and patient characteristics, leading to the surgical management of 63.7% of recurrences with a mean TTR was 4.3 years for surgically-managed recurrences. This TTR was significantly longer than in patients treated with radiation therapy. This may be explained by the management of meningioma recurrences, in which radiation therapy was proposed in some cases of recurrent meningiomas with documented growth but without clinical symptoms, whereas surgical treatment of recurrences was more often privileged in symptomatic patients or tumors with a higher WHO grade. The pertinence of a systematic post-operative adjuvant radiation therapy in intra-cranial meningiomas is actually evaluated in two ongoing randomized controlled trials designed to elucidate its relevance on clinicaltrials.gov (NCT03180268, NCT00895622), but the results thereof are not yet available.

Our study is the first to describe repeated surgeries for meningioma recurrences and the TTR between the different surgeries for recurrences of intra-cranial meningiomas. The TTR decreased significantly after first surgery (mean 4.3 years) and the second surgery (mean 2.5 years), was stable between the 3^rd^ and 4^th^ surgery, and was almost halved after the fourth, fifth and sixth surgery. This continuous and significant decrease of TTR between surgeries means that the benefit of surgical treatment of recurrences decreases with successive surgeries (Fig. 2). However, this decrease in TTR may also be the reflect of the decreased physical condition of the patient following the successive surgeries.

The meningioma’s WHO grade was identified as the main driver of recurrence, with a significantly higher proportion of WHO grade II and III meningiomas at recurrence. This highlights their more aggressive behavior and faster growth, resulting in a significantly shorter TTR, as previously described by Adegbite *et al*. and Ildan *et al*.^5,6^. However, our results are the first to show the strength of the effect of a high WHO grade effect on TTR after the 2^nd^ surgery and 3^rd^ surgery (OR 7.13 and 4.13 respectively).

Patient’s age was the sole patient-related statistically significant predictor of recurrence identified in our study. It was a protective factor of recurrence, a result which seems counter-intuitive, as older patients presents with more aggressive evolution of meningioma and show higher incidence of postoperative complications^20^. Surgery for a meningioma recurrence in the elderly may be a less pertinent option considering the shorter residual life expectancy combined the higher risk of complication in this population may tip the scale against a surgical treatment of meningioma recurrence considering the benefit-to-risk ratio. In this context, surgery may have been only proposed in selected cases in older patients inducing a selection bias^21,22^. Also, only surgically managed recurrences were considered in the analysis; data of patients with recurrence managed conservatively is missing. In our opinion, this may be why patient age is a statistically significant factor. Lastly, elderly patients have a shorter follow-up period since they are more at risk of death from other causes, an assumption further supported by the fact that patient age was a negative prognostic factor for OS in our cohort^19^.

In our cohort, the postoperative hematoma and infection rates remained stable whereas the rate of postoperative neurological worsening increased significantly, quadrupling between the first surgery and the recurrences.

The rate of postoperative hematomas was not increased after re-operations. This is in concordance with previous reports^23–28^. However, meningiomas have a higher risk of postoperative infection when compared to other intracranial tumors^29,30^.

The postoperative infection rate described in our cohort is lower than in previous published series (2.3% vs. 6–12%)^23,30^ and lower than the 16% rate reported in a series of recurrent meningiomas^10^. However, as superficial wound infections were often treated at local hospitals or in private practice, we considered in our study only postoperative infections requiring reoperations.

The rate of postoperative neurological worsening was lower in our cohort compared to the literature for the first surgery (3.9% vs. 10–14%)^23,26^. In contrast, the rate after the second and third surgery in our cohort was higher than the series reported in the literature (23.8% vs. 14%). This main be explained by the high proportion of skull-base meningiomas in our cohort (up to 50%), since these meningiomas represents an increased surgical challenge and an increased risk of postoperative morbidity in case of surgical re-treatment at the site of a previous surgery^10,28^.

The main strengths of this study are the clinical setting, the cohort’s size, and the length of follow-up (11 414 patient-years). Loss of follow-up was minimal (one patient moved abroad). All patients treated surgically for a meningioma during the inclusion and the follow-up period were included, minimizing the risk of an inclusion-bias.

These results based on an extensive cohort of Norwegian patients may not be extrapolated to other populations. The retrospective collection of data before 2003 and the association of retrospective and prospective patients in this cohort is a further limitation. The statistical analysis solely based on the 711 prospective patients identified 53 reoperation and 13 patients with a third surgery. Based on the prospective cohort, we confirmed the decreased TTR between surgeries but failed to identify predictive factors of recurrence due to a lack of statistical power (Supplementary Table and Fig. 1). We acknowledge that the association of prospective and retrospective data may be subject to question from a statistical point of view but we considered this an acceptable compromise to increase the size of the cohort and increase the statistical power of the analysis.

Because of the partly retrospective data collection, we do not dispose of precise data on germs for infections. Superficial surgical wound infections not requiring reoperations are excluded as they may have been treated in private practice outside of our neurosurgical center, and thus not referred to us may artificially lower our infection rate. Also, the partially retrospective collection of data led to some missing data limiting our analyses: postoperative neurological worsening being performed on a limited number of patients, no information on non-surgical complications such as deep venous thrombosis, no information on postoperative after the third surgery and not enough information to make the distinction between transient and permanent neurological deficit. The analysis of patient’s postoperative neurological status was limited due to incomplete data, since only 70% and 64% patients had complete documentation of post-operative complications after second and third surgery for recurrence, respectively. Also, data were limited to the date of surgery and the TTR for patients that underwent a fourth, fifth and sixth surgery.

## Conclusion

We observed that the TTR decreases significantly between surgeries in patients requiring repeated resections, indicating that surgical treatment of recurrences does not “reset the clock” but is indeed a “race against time”. This should be considered when assessing the benefit-to-risk ratio of patients undergoing repeated surgeries for a recurrent meningioma.

## Methods

### Patient cohort

A review of a Norwegian population-based cohort of intra-cranial meningiomas treated surgically at the Oslo University Hospital (OUH) was performed. OUH is a tertiary referral center composed of two neurosurgical units (Rikshospitalet and Ullevaal) covering altogether 3 million inhabitants and representing circa 56% of the Norwegian population. A total of 1469 consecutive patients were identified from a database (retro- and prospective inclusions from 1990 to 2002 and from 2003 to 2010, respectively). The characteristics of the cohort have been described in previously published reports^17,19,31–33^.

The surgical management aimed at achieving a complete tumor removal whenever possible, taking into consideration the patients’ and tumors’ characteristics. Based on the surgical report and the post-operative imaging, the EOR was assessed using the Simpson grade scale. Gross total resection (GTR) was defined as a Simpson grade I, II or III resection, according to the European Association of Neuro-Oncology (EANO)^2^.

Since the World Health Organization (WHO) criteria (previously benign, atypical or anaplastic) changed during the study period, the tumors in between 1990 to 2001 were reviewed by an expert neuropathologist and re-classified as WHO grade I (benign), WHO grade II (atypical) and WHO grade III (anaplastic). The histopathological diagnosis of all meningiomas included in this cohort was revised by an expert pathologist and reclassified according to the last WHO meningioma grading from 2016^3^. The distribution of the WHO grades was as follows: WHO I = 1352 (92.5%), WHO II = 77 (5.3%), and WHO III = 32 (2.2%), respectively.

The mean follow-up was 7.8 years ± 5.5 years (range 0–23 years), with a cumulated total of 11 414 patient-years follow-up. One patient moved abroad and was thus lost to follow-up.

All the recurring tumors with radio-clinical correlations, occurring at the site of the previous surgery were considered. In order to avoid subjectivity in differentiating postsurgical tumor remains from scars located near the resection sites, the TTR was delineated as the time between the first surgery and the first subsequent treatment (either radiotherapy or a new surgical procedure). Radiological recurrences without clinical expression, thus not requiring any adjuvant treatment were not considered. Tumors occurring at locations other than the primary site of the tumor were not considered.

Early post-operative complications were defined as post-operative on-site hematoma or surgical site infection requiring a second surgery, regardless of timeline, corresponding at least to a grade IIb complication, according to the Landriel-Ibañez classification^34^. All patients who underwent re-operation for hematoma evacuation or surgical site infection were included in this study. To assess the outcome, we reviewed the neurological status at last follow-up and compared it to the preoperative neurological status to identify any worsening of the postoperative neurological status.

Data on the time of surgery, the postoperative status and the occurrence of postoperative complications were consigned for the second and third surgery. Data were limited to the date of surgery and the TTR for patients that underwent a fourth, fifth and sixth surgery.

### Statistical analysis

Statistical analysis and graphical drawing were performed using R v3.5.1 (https://www.r-project.org). Statistical significance threshold was set at p = 0.05. Comparison of TTR between recurrences treated with surgery or radiation therapy, and the TTR between the different surgeries were performed using a Student t-test.

A multivariate analysis using a binomial general linearized model approach was performed to identify risk factors of recurrence after surgery. Statistically significant prognostic factors with an OR < 1 had a negative, and factors with an OR > 1 had a positive impact on recurrence risk.

### Ethics

The study was regulated by the Personal Data Act/Personal Health Data Filing System Act and approved by the Data Protection Official, registered Norwegian institutional review board at OUH (2017/5204). Under this regulation, patient’s written informed consent from was not required to collect and analyze data.

## Supplementary information

Supplementary data.
