# Supplementary material for: Benefits of re-do surgery for recurrent intracranial meningiomas
Source: Sci Rep. 2020 Jan 15;10:303. doi: 10.1038/s41598-019-57254-5 (PMC6962359; doi:10.1038/s41598-019-57254-5)
Supplement: Supplementary file 1 — Supplementary data. [file 41598_2019_57254_MOESM1_ESM.pdf]

Benefits of re-do surgery for recurrent intracranial meningiomas

Jean-Michel Lemée[1,2] MD, PhD, Marco V. Corniola[2,3] MD, Torstein R. Meling[2,3,4,5] MD, DPhil.

[1]Department of Neurosurgery, University Hospital of Angers, Angers, France

[2]Department of Clinical Neurosciences, Division of Neurosurgery, Geneva University Hospitals, Geneva, Switzerland

[3]Faculty of Medicine, University of Geneva, Geneva, Switzerland

[4]Department of Neurosurgery, Oslo University Hospital, Oslo, Norway

[5]Faculty of Medicine, University of Oslo, Oslo, Norway

Supplementary Table 1: Predictive factors of recurrence in the prospective part of the cohort (n=711)

NA: data not available; RT: radiation therapy; WHO: world health organization

	1st recurrence		2nd recurrence	
	**OR**	**p**	**OR**	**p**
**Age**	0.95 [0.92;1.01]	0.08	0.98 [0.97;1.01]	0.51
**Sex (Male)**	1.23 [0.64;2.31]	0.52	2.8 [0.27;2.14]	0.18
**Preoperative Karnofsky ≥ 70**	1.53 [0.43;9.84]	0.58	15.28 [0;∞]	0.99
**Skull base location**	1.90 [0.96;4.06]	0.07	0.24 [0.13;1.37]	0.13
**WHO grade**	6.13 [0.44;14.43]	0.42	0.01 [0; ∞]	0.99
**Simpson grade**	1.05 [0.78;1.38]	0.76	NA	NA
**Postoperative hematoma**	0.03 [0;39.70]	0.98	0.01 [0;∞]	0.99
**Postoperative infection**	2.11 [0.81;14.11]	0.13	0.01 [0; ∞]	0.99
**Postoperative radiation therapy**	0.56 [0.12;1.82]	0.38	NA	NA

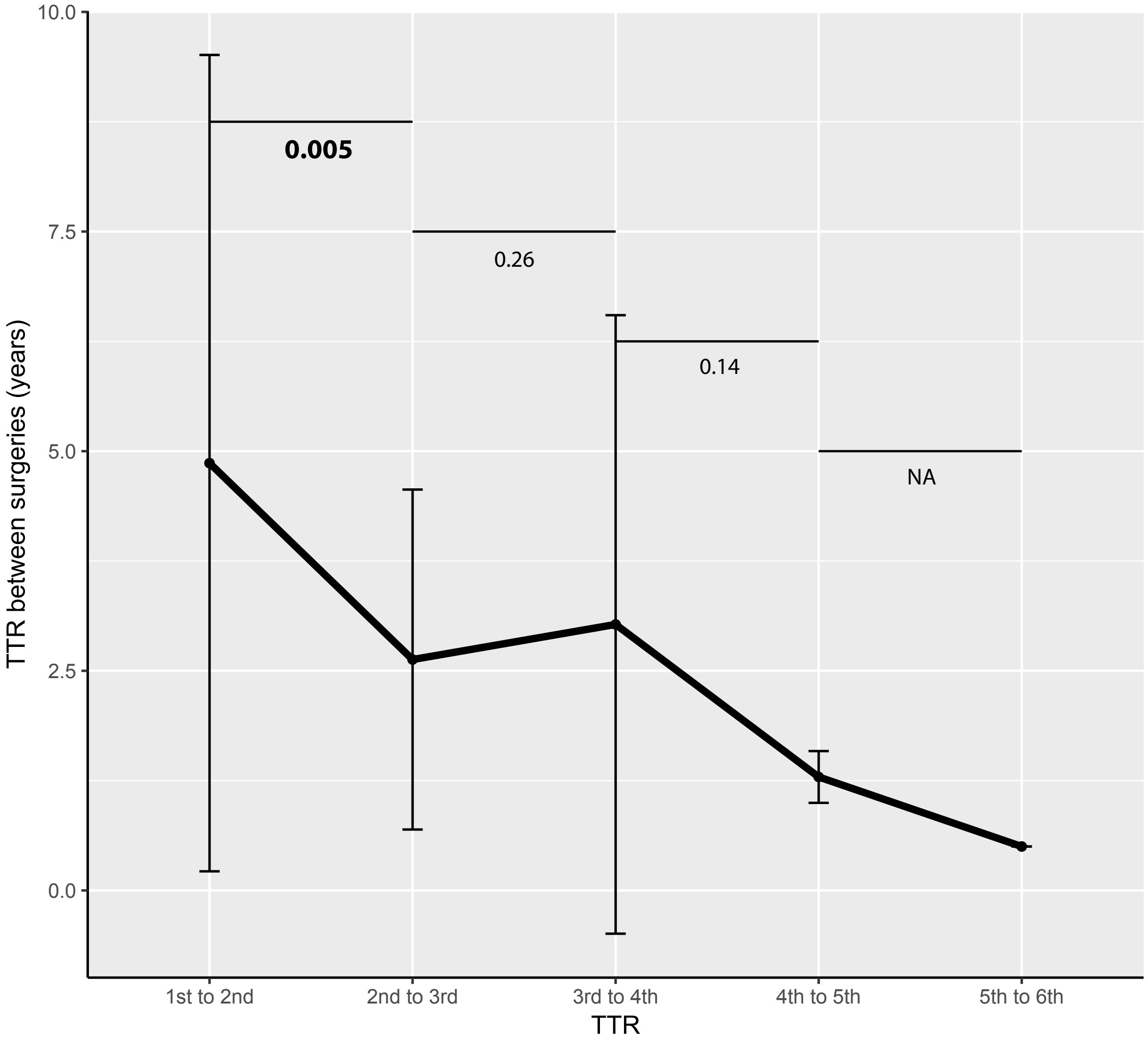

Supplementary Figure 1: Graphical representation of TTR between surgeries for recurrent meningioma in the prospective cohort (n=711)
N.A.: not available. No statistical test was performed since only 1 patient was surgically treated six times for a recurrent meningioma
